# The Incorrigible; a Demurrer

**Published:** 1905-10

**Authors:** 


THE INCORRIGIBLE; A DEMURRER.—The pharasaical editor of the *Octopus* practically pleads a demurrer. A wag of a lawyer here says: "In law, a demurrer is something like this: You bring suit against a neighbor to abate a nuisance you claim he has on his premises to your injury or detriment. You go in court and make the allegations. The other fellow says 'yes, you are right; all you say is true, but what are you going to do about it? You have no case, you can't help yourself.' That is a demurrer, and is often sustained by the court."

*The Octopus* has been arraigned for a nuisance, publishing the advertisements of *secret remedies,* denouncing the practice in others, yet shoving them under the nose of readers in every issue despite the calling down he had at Portland by resolutions introduced by "This here Jones" and others, resolutions calling on him to purge the paper of those ads, in which the formula is not given. He still publishes such ads. From the last issue, September 23d, I clip this: "For insomnia, Veronal, average dose, seven and one-half grains. Send for literature to————." While "Thigenol Roche" is recommended for acne, eczema, erysipelas, urticaria, rheumatism, scabies, burns, metritis, cervicitis, pruritus, salpingitis, hemorrhoids, etc., and the 'formula' is given thus: *"Sodium salt of the sulphonic acid of a synthetic sulpho oil."* How delightfully lucid that is. As clear as mud. Why, of course, everybody knows what a sodium salt of the sulphonic acid of a synthetic sulpho oil is. Oh, Simmons, Simmons, what a wag you are. I clip from page 936 of the *Journal,* A. M. A., September 23d, the following: After denouncing certain proprietary ads (which I have seen in the *Journal*), as nostrums, he says: "This brings up the problem we are trying to solve," viz.: "What is the difference between a 'secret proprietary medicine' advertised in medical journals to physicians, and a patent medicine advertised in newspapers to the public?"

Mr. Simmons has "solved the problem." He sees *no* difference; they are alike to him, and thus he is continuing to publish the advertisements of "secret remedies" all the same—because it pays—doubtless. If there is no difference, all should be excluded from the pages of the great "(mis)representative organ." But, as said before—"Yes, what you say is true, Mr. "Red Back," but what are you going to do about it?"

